# *Mycobacterium leprae* Phenolglycolipid-1 Expressed by Engineered *M. bovis* BCG Modulates Early Interaction with Human Phagocytes

**DOI:** 10.1371/journal.ppat.1001159

**Published:** 2010-10-21

**Authors:** Guillaume Tabouret, Catherine Astarie-Dequeker, Caroline Demangel, Wladimir Malaga, Patricia Constant, Aurélie Ray, Nadine Honoré, Nana Fatimath Bello, Esther Perez, Mamadou Daffé, Christophe Guilhot

**Affiliations:** 1 CNRS, IPBS (Institut de Pharmacologie et de Biologie Structurale), Toulouse, France; 2 Université de Toulouse, UPS, IPBS, Toulouse, France; 3 Institut Pasteur, Unité de Pathogénomique Mycobactérienne Intégrée, Paris, France; Weill Cornell Medical College, United States of America

## Abstract

The species-specific phenolic glycolipid 1 (PGL-1) is suspected to play a critical role in the pathogenesis of leprosy, a chronic disease of the skin and peripheral nerves caused by *Mycobacterium leprae*. Based on studies using the purified compound, PGL-1 was proposed to mediate the tropism of *M. leprae* for the nervous system and to modulate host immune responses. However, deciphering the biological function of this glycolipid has been hampered by the inability to grow *M. leprae in vitro* and to genetically engineer this bacterium. Here, we identified the *M. leprae* genes required for the biosynthesis of the species-specific saccharidic domain of PGL-1 and reprogrammed seven enzymatic steps in *M. bovis* BCG to make it synthesize and display PGL-1 in the context of an *M. leprae*-like cell envelope. This recombinant strain provides us with a unique tool to address the key questions of the contribution of PGL-1 in the infection process and to study the underlying molecular mechanisms. We found that PGL-1 production endowed recombinant BCG with an increased capacity to exploit complement receptor 3 (CR3) for efficient invasion of human macrophages and evasion of inflammatory responses. PGL-1 production also promoted bacterial uptake by human dendritic cells and dampened their infection-induced maturation. Our results therefore suggest that *M. leprae* produces PGL-1 for immune-silent invasion of host phagocytic cells.

## Author Summary

*Mycobacterium leprae*, the causative agent of leprosy, is a chronic human disease responsible for irreversible peripheral nerve damage and deformities. Lepromatous leprosy, the most severe form of the disease, is accompanied by T-cell unresponsiveness, suggesting that *M. leprae* has evolved strategies to modulate host immune responses. However, the molecular mechanisms of *M. leprae* infection remain poorly understood, mainly because this bacterium has been to date impossible to grow *in vitro*. The present study reports an innovative approach to study the contribution of a phenolic glycolipid (PGL-1) specific of *M. leprae* in the cross-talk of the pathogen with host cells. We reprogrammed a biosynthetic pathway in a surrogate host, *M. bovis* BCG, to make it synthesize and display PGL-1 in the context of a mycobacterial envelope. Using this novel microbial tool, we found that PGL-1 production enhances the cellular invasiveness of BCG and promotes the entry via complement receptor 3-mediated phagocytosis. Bacterial uptake via this route was associated with reduced inflammatory responses in infected human macrophages. In addition, we showed that PGL-1 production inhibited the infection-induced maturation of human dendritic cells. Our findings thus provide new insights into the contribution and molecular mechanisms of action of PGL-1 in leprosy pathogenesis.

## Introduction

Leprosy is a chronic human disease of the skin and peripheral nerves caused by the intracellular pathogen *Mycobacterium leprae*. Although control programs led by governmental and charitable agencies have reduced the number of patients from ∼10–15 million to less than 1 million over the last 10 years [1], the level of new cases persists at ∼250,000 per year in 2008 [2]. Therefore, in order to eradicate this disease, it is essential to assist multi-drug therapy programs with additional control strategies.

Lepromatous leprosy is the most severe manifestation of the disease and is characterized by poor cellular responses and uncontrolled proliferation of the bacilli throughout the skin. Lesions contain macrophages filled with bacteria, but few T lymphocytes and no organized granulomas [3], suggesting that *M. leprae* evades host immune recognition. Despite the early discovery of *M. leprae* in 1873, both the biology of this bacterium and the molecular basis of its pathogenicity remain obscure. Functional studies have been hampered by the incapacity to cultivate the leprosy bacillus *in vitro* and by its extremely slow growth in animal models (doubling time of ∼14 days). Among the molecules suspected to be critical for the pathogenesis of leprosy is the phenolic glycolipid 1 (PGL-1), a compound produced in large quantities by *M. leprae in vivo* [4]. PGL-1 consists of a lipid core formed by a long-chain β-diol, which occurs naturally as a diester of polymethyl-branched fatty acids. This core is ω-terminated by an aromatic nucleus that is glycosylated by a trisaccharide, which is highly specific of *M. leprae*. In contrast, the lipid core, called phenolphthiocerol dimycocerosates, is conserved in other mycobacterial species like *M. tuberculosis* and *M. bovis*, where it is linked to different species-specific saccharidic groups [5].

PGL-1 has attracted a lot of interest because it might represent a key virulence factor of *M. leprae*. Indeed, this compound is located at the outermost surface of *M. leprae* and therefore is ideally positioned to interact with host cell components. The trisaccharidic portion of PGL-1 was proposed to promote invasion of Schwann cells via binding to the G domain of the α2 chain of laminin-2 in the basal lamina, and may thus be responsible for the unique capacity of *M. leprae* to invade peripheral nerves [6], [7]. However, the critical importance of this interaction has been challenged by observations that mycobacteria unable to produce PGL-1 exhibited similar binding capacities to laminin-2 and Schwann cells [3], [8]. Therefore, the question of whether PGL-1 is the only determinant of *M. leprae* conferring tropism for peripheral nerves is still open. Supporting its putative involvement in the pathogenesis of the leprosy bacillus, Neill & Klebanoff have proposed that PGL-1 may be involved in the protection against oxygen radicals, as coating *Staphylococcus aureus* with purified PGL-1 or deacylated-PGL-1 increased its capacity to survive within human monocyte-derived macrophages and to resist *in vitro* to reactive oxygen species [9]. Consistent with these results, microbial glycolipids, including PGL-1, were found to be highly effective in scavenging oxygen radicals [10]. Whether endogenously expressed PGL-1 protects mycobacteria from the bactericidal mechanisms of host cells nevertheless remains to be established. Regarding the modulation of the host immune response, another major aspect of leprosy pathogenesis, several lines of evidence suggest that PGL-1 plays a critical role. First, PGL-1 purified from *M. leprae* was found to bind the complement component C3, thereby potentially promoting *M. leprae* uptake by phagocytes through complement receptors without triggering a strong oxidative burst [11]. Second, exogenously added PGL-1 modulated the cytokine response of human monocytes [12]. Third, *M. leprae* induced a poor activation and maturation of dendritic cells and dampened the T-cell responses induced by infected dendritic cells [13], [14]. This inhibition was partially relieved by treatment of *M. leprae*-infected cells with anti-PGL-1 antibodies [13]. Together, these studies suggested that PGL-1 is a major virulence factor of *M. leprae*. However, the cellular and molecular mechanisms by which PGL-1 participates in the cross-talk between the pathogen and the host cells remain unclear. Clearly, tools to address these questions were missing.

To our best knowledge, the lipid constituents of the *M. leprae* cell envelope are structurally almost identical to those of *M. bovis* BCG, except PGL. Importantly, in contrast to *M. leprae*, *M. bovis* BCG can be cultivated *in vitro* and molecular tools are available to modify its genome. Therefore, *M. bovis* BCG reprogrammed to synthesize PGL-1 constitutes an ideal surrogate organism to investigate the physiological role of this molecule in *M. leprae* pathogenicity.

Here, we have identified the *M. leprae* genes required for the biosynthesis of the trisaccharidic domain of PGL-1 and we have genetically engineered *M. bovis* BCG to make it synthesize and export PGL-1. Using this recombinant strain, we studied the impact of PGL-1 on the initial encounter of mycobacteria with human phagocytes. We found that PGL-1 deviates the route of mycobacterial entry into human macrophages and dendritic cells to suppress the initiation of innate immune responses.

## Results

### Identification of candidate genes for the synthesis of the saccharidic domain of PGL-1

**Figure 1:**
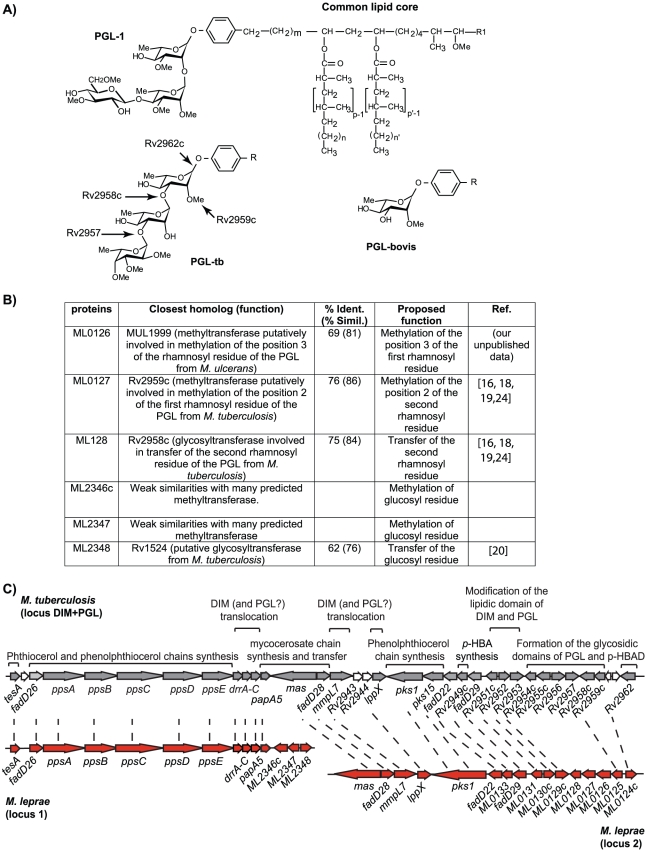
Identification of the genes involved in the formation of the saccharidic domain of PGL-1 from *M. leprae*. (A) Structure of the PGL from *M. leprae*, *M. tuberculosis* and *M. bovis* and role of the various enzymes from *M. tuberculosis* in the formation of the saccharidic domain of PGL-tb. In *M. leprae*, p, p′ = 4; n, n′ = 16–20; m = 17 [5]; R1 = −CH_2_−CH_3_ or −CH_3_; R = common lipid core. (B) Candidate proteins for the formation of the terminal disaccharide of PGL-1 and proposed enzymatic function. (C) DIM+PGL loci in *M. tuberculosis* and in *M. leprae*. Orthologs are linked by dashed lines. Known functions of the encoded proteins are indicated above the open-reading frames.

The structure of the main PGL produced by *M. bovis* BCG consists of phenolphthiocerol dimycocerosates glycosylated at the ω-terminus by a 2-*O*-methylrhamnose (Figure 1A). The lipid core is structurally identical to that of the PGL-1 from *M. leprae*, but in PGL-1 the saccharidic domain is 3,6-di-*O*-Me-Glcp (β1->4) 2,3 di-*O*-Me-Rhap (α1->2) 3-*O*-Me-Rhap (α1- linked to phenol ring) (Figure 1A) [15]. Therefore, to reprogram the PGL biosynthesis pathways of *M. bovis* BCG to produce PGL-1, we needed (i) to prevent the methylation at position 2 of the first rhamnosyl residue, (ii) to provide *M. bovis* BCG with the enzymes required for methylation at position 3 of the first rhamnose, and for synthesis and transfer of the terminal disaccharide on position 2. The Rv2959c methyltransferase responsible for methylation of position 2 of the first rhamnosyl residue in the PGL of *M. tuberculosis* has been identified [16]. By analogy with *M. tuberculosis*, inactivation of this gene in *M. bovis* BCG was expected to result in the production of unmethylated PGL, the starting point of our reprogramming process. The next step was to identify the *M. leprae* genes required for the transfer of the terminal disaccharide and methylation at the defined positions of the carbohydrate extension. We reasoned that six enzymes would be required: two glycosyltransferases and four methyltransferases, assuming that the same enzyme methylates position 3 on the both rhamnosyl units. Having shown previously that genes involved in the biosynthesis and translocation of lipids in mycobacteria are usually clustered in the genome [17], we performed bioinformatic analyses of the *M. leprae* genome using the following criteria: genes encoding proteins with similarities to known glycosyl- or methyltransferases, clustering of these genes within the *M. leprae* genome, and proximity to orthologs of known PGL biosynthetic genes. Using this strategy, we identified 6 candidates for the methylation and transfer of the two terminal residues and for the methylation of the first rhamnosyl residue: *ML0128* and *ML2348* encoding proteins with similarities to glycosyltransferases, and *ML0126*, *ML0127*, *ML2346c* and *ML2347* encoding proteins with similarities to methyltransferases (Figures 1B and 1C) [18], [19]. The six candidate genes were clustered on two genome regions containing orthologs of genes involved in the formation of *M. tuberculosis* PGL (PGL-tb) and the related phthiocerol dimycocerosates (Figure 1C). In *M. tuberculosis* [20], these genes map to a single locus that appears to be divided in *M. leprae*. Sequence similarities between the proteins encoded by the candidate genes and other enzymes allowed us to assign them a putative function (Figure 1B).

### Construction of recombinant BCG producing PGL-1

**Figure 2:**
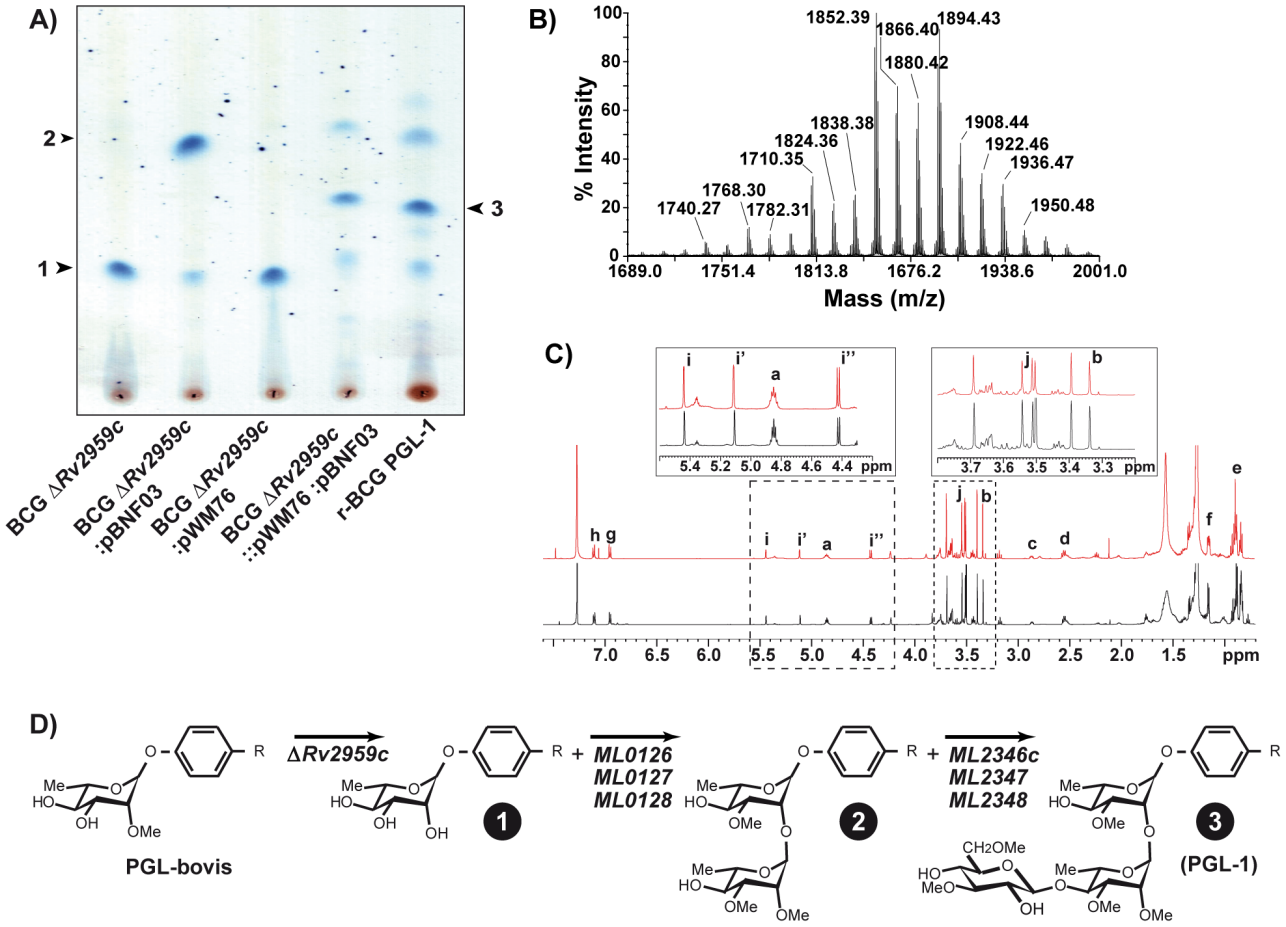
Construction of a recombinant BCG producing the PGL-1 of *M. leprae*. (A) TLC analysis of lipids extracted from various recombinant BCG strains. Lipid extracts dissolved in CHCl_3_ were run in CHCl_3_/CH_3_OH (95∶5, v/v). Glycolipids were visualized by spraying the plates with 0.2% anthrone (w/v) in concentrated H_2_SO_4_. The various glycolipids analyzed by mass spectrometry and ^1^H NMR are numbered. (B) MALDI-TOF mass spectrum of purified PGL-1 produced by r-BCG PGL-1. (C) ^1^H NMR spectra of native PGL-1 (black spectrum) from *M. leprae* and recombinant PGL-1 (red spectrum) from r-BCG PGL-1. Inserts correspond to enlargement of parts of spectra that are relevant for structure determination. (D) Structures of the glycolipids produced by the various recombinant BCG strains after deletion of *Rv2959c* gene and transfer of 3 or 6 *M. leprae* genes.

To reprogram the PGL biosynthesis pathway in *M. bovis* BCG, we first disrupted the *Rv2959c* ortholog by allelic exchange [21]. One clone exhibiting the expected PCR profile for a BCG *ΔRv2959c::km* mutant was retained for further studies (Figure S1). The kanamycin cassette used in this construct, flanked by two *res* sites from transposon γδ, was removed after transient expression of the transposon γδ resolvase from plasmid pWM19 [22] to generate the unmarked BCG *ΔRv2959c* (Figure S1). The lipids produced by this mutant strain were analyzed by thin layer chromatography (TLC) (Figure 2A). As expected, the spot corresponding to PGL-bovis was no longer detectable and a new, more polar, glycolipid (product 1) was observed. Matrix-assisted laser desorption-ionisation time-of-flight (MALDI-TOF) mass spectrometry analyses of purified product 1 gave a series of pseudomolecular ions (M+Na)^+^ with a major peak at 1516 amu, *i.e.* 14 mass units lower than those of the usual PGL from wild-type (WT) *M. bovis* BCG [23]. Therefore, we concluded that this compound corresponded to the expected unmethylated rhamnosyl-phenolphthiocerol dimycocerosates.

Next, a DNA fragment encompassing the *ML0126*, *ML0127* and *ML0128* genes was inserted into the mycobacterial vector pMIP12H [24] to yield plasmid pBNF03 (Figure S1). In parallel, a second DNA fragment carrying *ML2346c*, *ML2347* and *ML2348* genes was inserted into the integrative vector pMV361 [25] to give pWM76 (Figure S1). These two constructs were transferred independently or simultaneously into the BCG *ΔRv2959c* mutant. Lipids were extracted from BCG *ΔRv2959c*:pBNF03, BCG *ΔRv2959c*::pWM76 and BCG *ΔRv2959c*:pBNF03::pWM76 and analyzed by thin-layer chromatography (TLC) (Figure 2A). In the case of BCG *ΔRv2959c*::pWM76, no new glycolipid was detected. In sharp contrast, a new glycolipid, product 2, exhibiting higher mobility was detected in extracts from *M. bovis* BCG *ΔRv2959c*:pBNF03. MALDI-TOF mass spectrometry analysis of product 2 gave a series of pseudomolecular ions (M+Na)^+^ centered at *m/z* 1704 consistent with the addition of a deoxyhexosyl residue and three *O*-Methyl groups to product 1 produced by BCG *ΔRv2959c*. These results strongly supported the hypothesis that *ML0126*, *ML0127* and *ML0128* genes are involved in the transfer of the second rhamnosyl unit and the methylation of the first two sugar residues of PGL-1 in *M. leprae*. When the two plasmids pBNF03 and pWM76 were transferred into BCG *ΔRv2959c*, at least three glycolipids were detected (Figure 2A). The two quantitatively minor compounds exhibited the same mobility than the PGL-1 intermediates observed in BCG *ΔRv2959c* and BCG *ΔRv2959c*: pBNF03. When analyzed by MALDI-TOF mass spectrometry, the most abundant compound, product 3, showed a series of pseudomolecular ions (M+Na)^+^ with a major peak at *m/z* 1894 in agreement with the addition of a di-*O*-Me-hexosyl unit (190 uma) to the PGL-1 intermediate, product 2.

These results suggested that the six proteins encoded by genes *ML0126*, *ML0127*, *ML0128*, *ML2346c*, *ML2347* and *ML2348* are sufficient to produce the specific saccharidic domain of PGL-1. The six genes were grouped in a single integrative plasmid, named pWM122 (Figure S1). This plasmid was transferred into BCG *ΔRv2959* to yield r-BCG PGL-1. PGL-1 production by the recombinant strain was confirmed by TLC analysis, MALDI-TOF mass spectrometry (Figure 2B) and NMR spectroscopy analysis (Figure 2C). When analyzed by mass spectrometry, product 3 purified from r-BCG PGL-1 showed a series of pseudomolecular ions (M+Na)^+^ with a major peak at *m/z* 1894 in agreement with the expected structure (Figure 2B). The characterization of the saccharidic part was achieved by NMR spectroscopy analysis, using PGL-1 from *M. leprae* as reference. The two ^1^H-NMR spectra were super imposable (Figure 2C). All the signals unambiguously reflecting the presence of phenolphthiocerol dimycocerosates were seen in the spectrum of product 3: proton resonances of *p*-substituted phenolic group (signals **g**, **h** at 6.95 and 7.14 ppm), of methine of the esterified β-glycol (**a**, 4.85 ppm), of methyl substituents of polymethyl-branched fatty acids (**e**, 0.8–1 ppm; **f**, 1.15 ppm), of methoxyl and methine groups on the phthiocerol (**b**, 3.33 ppm and **c**, 2.85 ppm). The presence of the three de-shielded anomeric protons confirmed the presence of a trisaccharidyl part in product 3. The signals **i**, **i′** and **i″** at 5.43 ppm, 5.12 ppm and 4.42 ppm were assigned respectively, to the resonances of anomeric protons of 3-*O*-Me rhamnosyl, 2,3-di-*O*-Me rhamnosyl and 3,6-di-*O*-Me glucosyl residues [15]. In addition, five singlets were observed in the region of sugar-linked methoxyl (OMe) proton resonances at 3.35–3.7 ppm whose chemical shift values were identical to those found for PGL-1. All these results identified product 3 as PGL-1 and demonstrated the role of the six transferred genes from *M. leprae* in the formation of the saccharidic domain of PGL-1 (Figure 2D).

### Characterization of the recombinant BCG strain producing PGL-1

We first compared the amounts of PGL produced by WT BCG and r-BCG PGL-1 in liquid culture. Each strain was cultured to exponential phase in liquid medium and PGL were labeled for 24h with [1-^14^C] propionate, a precursor known to be incorporated in methyl-branched fatty acids containing lipids, such as PGL. Analysis of the labeled lipids by TLC showed that both strains produced comparable amounts of PGL, with PGL-1 accounting for approximately 20% of the total PGL in r-BCG PGL-1 after 24h. As *M. leprae* cannot be cultivated *in vitro*, we compared the amounts of PGL-1 produced by r-BCG PGL-1 and *M. leprae* by analyzing on TLC similar quantities of total lipids extracted from *in vitro* grown r-BCG PGL-1 and WT *M. leprae* obtained from infected armadillos. The amount of PGL-1 found in r-BCG PGL-1 was approximately 2-fold lower than that found in *M. leprae*. As observed in Figure 2A and in the labeling experiments (data not shown), several biosynthetic intermediates were found in lipid extracts of r-BCG PGL-1. Interestingly, some of these intermediates were also found in *M. leprae* extracts but in lower quantities [26]. One possible explanation for the occurrence of significant amounts of biosynthetic intermediates in the recombinant BCG strain may reside in the fact that *M. leprae* genes were not optimally expressed in *M. bovis* BCG, possibly due to their lower GC content. Another explanation might be the different growth conditions used (*in vivo* in infected armadillos for *M. leprae* and *in vitro* for r-BCG PGL-1 and WT BCG). Indeed, we observed that the use of Sauton medium instead of 7H9 to grow r-BCG PGL-1, led to production of higher proportion of PGL-1 (data not shown).

**Figure 3:**
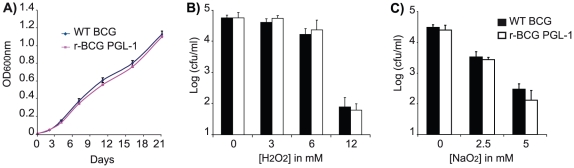
Microbiological properties of recombinant BCG. A) *In vitro* multiplication of WT BCG (blue line) and r-BCG PGL-1 (magenta line). 3-week cultures of both BCG and r-BCG PGL-1 were diluted to final OD_600nm_ = 0.001 in 7H9 broth containing ADC and 0.05% Tween 80 and incubated at 37°C. At the indicated time points, the OD_600nm_ was measured. Resistance to H_2_O_2_ (B) or NaNO_2_ (C). Exponentially growing WT BCG and r-BCG PGL-1 were diluted 1∶100 in 7H9 broth supplemented with ADC and containing various concentrations of H_2_O_2_ (0, 3, 6 or 12 mM) and NaNO_2_ (0, 2.5 or 5mM). For NaNO_2_, pH was adjusted to 5.5. After 24 h of incubation with ROI or RNI, serial dilutions were plated on 7H11 supplemented with OADC and cfus were evaluated after 3 weeks of incubation at 37°C. The indicated values are means (+/− SEM) of three independent experiments.

Having modified seven enzymatic steps in BCG, we next evaluated whether this metabolic reprogramming interfered with some basic microbiological properties of BCG. No difference in colony morphology or colony size could be detected in r-BCG PGL-1 following growth on Petri plates, compared to the WT control (data not shown). Moreover, the growth curves of both strains in liquid medium were super-imposable during the 3-weeks observation period (Figure 3A). Since PGL-1 was proposed to confer protection against reactive oxygen species, we also compared the viability of WT BCG and r-BCG PGL-1 exposed for 24 h to increasing concentrations of hydrogen peroxide (H_2_O_2_) or sodium nitrite (NaNO_2_) (at pH 5.5) (Figure 3). Although H_2_O_2_ and NaNO_2_ efficiently reduced bacterial viability at concentrations higher than 6 mM and 2.5 mM respectively, PGL-1 production did not modify the cell resistance to reactive oxygen or nitrogen intermediates (Figure 3B–C).

Together, these results suggested that basic microbiological properties of BCG such as colony morphology, growth rates or stress resistance were not affected by the metabolic reprogramming.

### PGL-1 production augments BCG infectivity and intracellular growth in human macrophages

**Figure 4:**
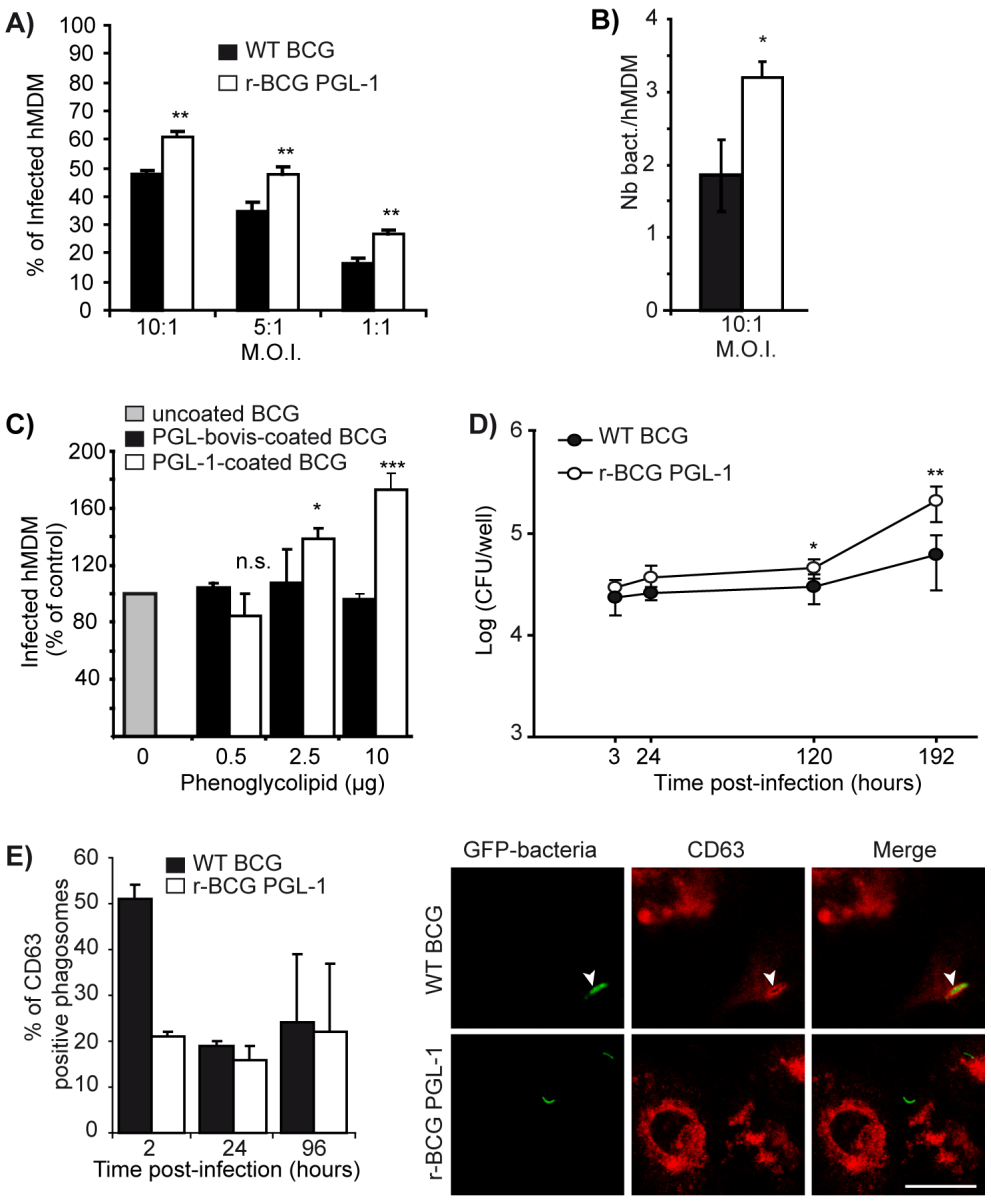
Effect of PGL-1 production on hMDM infection. (A) Percentage of infected hMDM after 2 hours of contact with the mycobacterial strains at various MOI. (B) Number of bacteria per hMDM after 2 hours of contact with WT BCG or r-BCG PGL-1 at MOI 10∶1. (C) Percentage of infected macrophages following contact with uncoated WT BCG (grey bar), WT BCG coated with PGL from *M. bovis* (black bars) or with PGL-1 from *M. leprae* (white bars) at MOI 10∶1. 100% corresponds to 26% of infected macrophages. (D) Intracellular multiplication of WT BCG and r-BCG PGL-1 in hMDM. hMDM were infected for 2h at MOI 10∶1 and the number of cfus was evaluated at various time points by plating serial dilutions of cell lysates on mycobacterial solid medium. (E) Colocalization of WT BCG or r-BCG PGL-1 with CD63 at 2h, 24h and 96h post infection. Representative confocal microscopy images of WT or r-BCG PGL-1 phagosomes within CD63-immunostained macrophages at 2h post-infection. White arrows indicated a phagosome counted positive for CD63. Bar: 4µM. The percentage of phagosomes positive for each marker was calculated by counting 100 phagosomes from at least 10 different fields in duplicate. The values are means ± SEM of 4 independent experiments for panels A, and 2 independent experiments for panels B, C, D and E each performed in triplicate or duplicate for panel E. Black bars correspond to BCG control and white bars to r-BCG PGL-1. The significance of differences between BCG control and r-BCG PGL-1 was evaluated: *, p<0.05 ;**, p<0.01; ***, p<0.001; n.s., not significant.

We then used r-BCG PGL-1 to investigate the role of PGL-1 in host cell infection. For this purpose, fluorescent forms of the WT and recombinant BCG strains were constructed by transferring a replicative plasmid carrying the *gfp* gene under the control of a mycobacterial promoter. We first compared r-BCG PGL-1 and the parental BCG strain for their capacity to invade human monocyte-derived macrophages (hMDM), or human dendritic cells (hDC), as these cell populations play major roles in the initiation and regulation of inflammatory responses. Strikingly, the number of hMDM infected by r-BCG PGL-1 was increased by 30±6% compared to that infected by parental BCG after 2 hours of interaction under non-opsonic conditions. This difference was observed for all the multiplicity of infection (MOI 10 to 1) tested (Figure 4A). Moreover, the number of intracellular r-BCG PGL-1 was increased by 70% compared to WT BCG (3.2+/−0.22 versus 1.85+/−0.5 bacilli/cell) at the analyzed MOI 10∶1 (Figure 4B). Opsonisation of the bacilli by pre-incubation with human serum markedly augmented the phagocytosis of both strains by hMDM to reach 205±9% and 218±8% for WT BCG and r-BCG PGL-1 respectively at MOI 10 (when normalized to 100% for BCG under non-opsonic condition). However, it abolished the difference previously observed between WT BCG and r-BCG PGL-1. Similar observations were made with hDC, *e.g.* enhanced uptake of r-BCG PGL-1 compared to WT BCG under non-opsonic conditions (Figure S2). As for hMDM infection, addition of human serum abolished the difference between WT BCG and r-BCG PGL-1 (data not shown).

To determine if this effect was directly related to the presence of PGL-1 at the surface of r-BCG PGL-1, purified PGL-1 or PGL-bovis were adsorbed onto WT BCG and the invasion efficiency of coated and uncoated strains were compared (Figure 4C). Adsorption of purified PGL-1 onto WT BCG increased its capacity to invade hMDM in a dose-dependent manner when compared to the uncoated strain (Figure 4C). In contrast, the coating of WT BCG with PGL-bovis had no significant effect on bacterial internalization (Figure 4C). Together, these results clearly established that, in the absence of opsonin, surface-exposed PGL-1 significantly enhances the bacterial infectivity.

Having shown that PGL-1 promotes host cell invasion, we then examined whether WT BCG and r-BCG PGL-1 differed in their capacity to multiply within hMDM. The intracellular loads of WT BCG and r-BCG PGL-1 were evaluated over a 8-day period by counting the intracellular colony forming units (cfus) at various time-points post-infection. As depicted in Figure 4D, the number of cfus was higher for r-BCG PGL-1 at every time point due to the enhanced invasion efficiency. In addition, the intracellular growth of r-BCG PGL-1 was superior to that of WT BCG with a two-fold and four-fold higher cfu count at 4 days and 8 days post-infection, respectively.

To determine if the growth advantage of r-BCG PGL-1 was associated with altered phagosomal maturation toward fusion with lysosomes, bacilli-containing phagosomes of hMDM infected with either WT BCG and r-BCG PGL-1 were compared for their acquisition of maturation markers. No difference in phagosome staining with lysotracker, v-ATPase, or CD63 could be detected between WT BCG and r-BCG PGL-1 at 24h and 96h, indicating that the maturation of phagosomes containing either WT BCG or r-BCG PGL-1 was not dramatically changed (Figure 4E and data not shown). However, at 2h post-infection, the number of CD63-positive phagosomes was significantly higher in cells infected with WT BCG, compared to cells infected with r-BCG PGL-1 (Figure 4E). This difference was not retained at later time points, suggesting that, although the phagosome maturation was not affected by the occurrence of PGL-1 at the surface of mycobacteria, the initial bacilli-containing vacuole was not exactly the same for WT BCG and r-BCG PGL-1.

### PGL-1 promotes the entry of r-BCG into human macrophages via CR3-mediated phagocytosis

**Figure 5:**
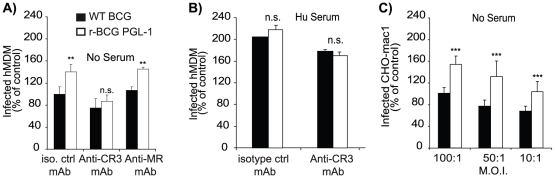
Role of various receptors in the enhanced invasiveness of human macrophages by r-BCG PGL-1. (A) Effect of a pre-incubation of hMDM with antibodies blocking either CR3 or MR, or irrelevant isotype controls, on the percentage of infected hMDM. (B) Effect of pre-incubation of hMDM with human serum and a blocking anti-CR3 mAb, or an irrelevant isotype control antibody, on the percentage of infected cells. (C) Percentage of infected CHO-Mac1 cells after an overnight contact with bacteria at various MOI. Data are presented as the percentage of phagocytosis with respect to WT BCG under non-opsonic conditions (100%). 100% corresponded to 34% of infected cells for panel A and B, and 38% for panel C. The values are means ± SEM of 3 or 4 independent experiments. Black bars corresponded to BCG control and white bars to r-BCG PGL-1. The significance of differences between BCG control and r-BCG PGL-1 was evaluated:**, p<0.01; ***, p<0.001; n.s., not significant.

Our results established that r-BCG PGL-1 infected hMDM more efficiently than WT BCG *via* a route leading to poor early acquisition of CD63. Since complement receptor 3 (CR3) and mannose receptor (MR) mediate the non-opsonic internalization of several mycobacterial species, such as *M. kansasii* [27], *M. tuberculosis* [28] or *M. leprae* [29], we assessed the possible involvement of these receptors in the uptake of r-BCG PGL-1. The effect of a pre-treatment with blocking antibodies raised against human CR3 or MR on the differential uptake of WT BCG and r-BCG PGL-1 by hMDM was evaluated. Anti-CR3 blocking antibodies slightly, but not significantly, modulated the phagocytosis of WT BCG (25%±17% inhibition with anti-CR3) (Figure 5A). Phagocytosis of WT BCG was not affected by the anti-MR antibody (Figure 5A). In contrast, a marked inhibition of r-BCG PGL-1 uptake by hMDM (54±11% inhibition, p<0.01) was observed following pre-incubation with an anti-CR3 antibody (Figure 5A). CR3 blockade restored r-BCG PGL-1 phagocytosis rates similar to those observed with WT BCG (Figure 5A). This effect was specific of the anti-CR3 antibody since it was not observed in the presence of the anti-MR or isotype control antibodies. In the presence of fresh human serum, the uptake of r-BCG PGL-1 was similar to that of WT BCG and superior to that of non-opsonized bacteria (Figure 5B). Pre-treatment with an anti-CR3 antibody reduced the uptake of both r-BCG PGL-1 and WT BCG to a similar extent (Figure 5B). Together, these results strongly suggested that PGL-1 expression confers on BCG the capacity to exploit the CR3 pathway for hMDM invasion in non-opsonic conditions.

To investigate further this hypothesis, we evaluated the differential capacity of WT BCG or r-BCG PGL-1 to infect recombinant CHO cells expressing human CR3 (CHO-Mac1) [30]. Following overnight incubation with mycobacteria at MOI (100∶1), only 5 to 7% of control CHO cells had ingested at least one bacterium under non-opsonic conditions and up to 10% in the presence of fresh serum. No difference between the WT BCG and r-BCG PGL-1 was observed. Expression of human CR3 by CHO cells resulted in enhanced mycobacterial uptake, with up to 34±7% of CHO-Mac1 cells infected with WT BCG. These results showed that WT BCG may use to some extent the CR3 pathway to invade phagocytes. However, as indicated by the poor inhibition of WT BCG uptake by hMDM treated with anti-CR3 antibody, BCG preferentially employs other routes for macrophage invasion. Importantly, uptake of r-BCG PGL-1 by CR3-expressing CHO was much more important than that of WT BCG, irrespectively of the MOI (Figure 5C). In accordance with our previous findings, opsonic conditions completely abolished the difference between WT BCG and r-BCG PGL-1 (data not shown).

Collectively, these results demonstrated that PGL-1 improves mycobacterial entry into hMDM via the CR3 pathway, a route poorly accessible for WT BCG in the absence of opsonins.

### PGL-1 production impairs the initiation of innate immune responses

**Figure 6:**
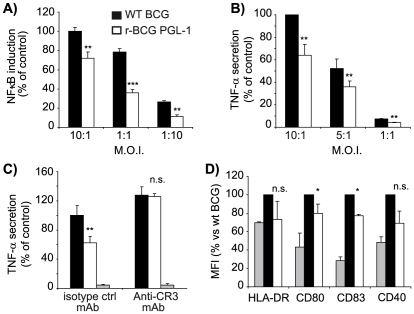
PGL-1 production impairs the initiation of innate immune responses. (A) Activation of the NF-κB pathway, as evaluated by the THP-1 Blue cell line and assay of SEAP activity, following infection with WT BCG (black bars) or r-BCG PGL-1 (white bars) at various MOI. The mean OD_630_nm values for WT BCG were 1.13, 0.905, and 0.246 at MOI 10∶1, 1∶1 and 1∶10 respectively whereas the value obtained for r-BCG PGL-1 were 0.87, 0.368 and 0.073 at the same MOI. The value used to normalize at 100% was 1.13 units. (B) TNF-α production by hMDM after 2 hours of post-infection. One hundred percent corresponded to 10538±1579 pg/ml. (C) TNF-α production by hMDM pre-incubated with mAbs directed against CR3 or with irrelevant isotype control and infected during 2 hours at a MOI of 10∶1. One hundred percent corresponded to 4101±551 pg/ml. (D) Expression of maturation markers at the surface of infected hDC, as analyzed by flow cytometry. Data are presented as the percentage of SEAP activity, TNF-α production or MFI with respect to the WT BCG (100%). The values are means ± SEM of 2 (panels A and C) and 4 independent experiments (panels B and D). In panels C and D, grey bars corresponded to uninfected controls. Differences between BCG control and r-BCG PGL-1 were statistically evaluated: *, p<0.05 ;**, p<0.01; ***, p<0.001; n.s., not significant.

We next examined whether PGL-1 production influenced the innate responses of human phagocytes to mycobacterial infection. This was first investigated by monitoring the effect of PGL-1 on the activity of the transcription factor NF-κB, which controls the expression of multiple inflammatory genes in hMDM and hDC. We used a THP-1 cell line transfected with a reporter system under the control of a promoter inducible by NF-κB. Infection of THP-1 cells with WT BCG induced strong expression of the reporter gene, indicative of potent activation of the NF-κB pathway. After 16 h of incubation in the detection medium, the mean OD_630_nm values obtained for the WT BCG were 23%, 60%, 70% higher than for r-BCG PGL-1 at MOI 10∶1, 1∶1 and 1∶10, respectively (Figure 6A). Therefore, the NF-κB response triggered by r-BCG PGL-1 was significantly lower (p<0.01) than that of WT BCG, whatever the MOI considered. Accordingly, hMDM infected with r-BCG PGL-1 produced lower amounts of the inflammatory cytokine TNF-α than BCG-infected controls after 2 hours of infection, even though bacteria expressing PGL-1 were more efficiently internalized than the WT controls (Figure 6B). Other cytokines, IL-12 (p40 and p70) and IL-10, were also assayed. Both WT BCG and r-BCG PGL-1 induced the production of poor levels of these cytokines both at 2h and 24h post-infection, and no difference was observed between WT-BCG and r-BCG PGL-1 infected hMDM (Figure S3).

To evaluate if the route of r-BCG PGL-1 entry into hMDM could explain the defective TNF-α production by infected hMDM, we examined the level of production of this cytokine when CR3-mediated phagocytosis was blocked. Incubation of hMDM with an anti-CR3 antibody, or an irrelevant isotype matched antibody did not trigger any significant TNF-α secretion. As expected, the presence of the control antibody did not affect the difference observed between WT BCG and r-BCG PGL-1: infection of hMDM with r-BCG PGL-1 resulted in defective TNF-α production, compared to hMDM infected with WT BCG (Figure 6C). In contrast, hMDM infected with BCG or r-BCG PGL-1 in the presence a blocking anti-CR3 antibody produced equivalent levels of TNF-α, demonstrating the critical role of CR3 in the down modulation of the inflammatory response induced by r-BCG PGL-1 (Figure 6C).

In parallel, we evaluated by flow cytometry the effects of PGL-1 on the phenotypic maturation of hDC following infection with WT BCG or r-BCG PGL-1. Here, only cells harboring fluorescent bacilli were considered, and propidium iodide (PI)^+^ hDC were excluded from the analysis. Notably, a reproducible inhibition of maturation was observed in hDC infected with r-BCG PGL-1 compared to BCG-infected hDC, as witnessed by the reduced surface expression of MHC class II, CD80, CD83 and CD40 (Figure 6D).

From these results, we conclude that bacterial production of PGL-1 suppresses the initiation of innate immune responses by infected phagocytes. In the case of hMDM, the immunomodulatory effects of PGL-1 are due to the preferential use of the CR3 pathway for bacterial phagocytosis.

## Discussion

In this study, we successfully modified the PGL biosynthetic pathway in BCG to generate a recombinant strain expressing PGL-1, thereby circumventing the difficulties in growing and genetically manipulating *M. leprae*. Like the native molecule in the leprosy bacillus, BCG-expressed PGL-1 was located in the outermost layer of the envelope (data not shown). Since both species have otherwise very similar envelopes, our r-BCG PGL-1 strain represented an ideal surrogate of *M. leprae* for studying PGL-1 interactions with host cells in a relevant biochemical and structural context. We found that PGL-1 promoted bacterial entry in phagocytes *via* CR3, a property not shared by the phenolic glycolipids of other mycobacterial species such as *M. bovis*. Importantly, deviation of the phagocytosis pathway resulted in reduced innate immune responses and was associated with improved intracellular multiplication. Our findings thus strongly suggest that *M. leprae* has evolved PGL-1 production as a strategy to escape innate immunity and establish long-term residence in the host.

The biosynthesis pathway of PGL involves more than 20 enzymatic steps. The steps required for the formation of the lipid core are common to all mycobacterial species producing PGL, and orthologs of the required genes could be identified by genome comparisons. In contrast, the saccharide appendage of PGL is species-specific. In the present study, we report the identification of the genes of *M. leprae* that are necessary and sufficient for its synthesis. Our bioinformatic analyses and the finding that the transfer of three *M. leprae* genes (*ML0126*, *ML0127* and *ML0128*) leads to the production of a PGL-1 intermediate harboring 2,3 di-*O*-Me-Rhap (α1->2) 3-*O*-Me-Rhap domain, led us to propose that: i) ML0128 is the rhamnosyl transferase involved in the attachment of the second rhamnosyl residue on position 2 of the first unit, ii) ML0127 is the methyltransferase involved in the methylation of position 2 of the second rhamnosyl residue, iii) and ML0126 is the enzyme responsible for methylation at position 3 of the first and second sugar residues. With regard to the transfer and modification of the terminal glucosyl unit, we concluded that ML2348 is the glucosyltransferase and ML23246c and ML2347 are the methyltransferases required for the modification of the 3 and 6 positions.

We investigated the role of PGL-1 in the early steps of mycobacterial interaction with host immune cells and found that PGL-1 augments the capacity of recombinant BCG to invade phagocytes, improves the multiplication of mycobacteria in infected hMDM, and impairs the infection-induced inflammatory responses. Uptake of BCG by macrophages occurs *via* various receptors including CR3 [31], [32]. Nevertheless, CR3 is poorly used by human macrophages to internalize BCG and needs to be activated, notably through cooperation with other cell surface receptors, such as CD14/TLR2, for efficient phagocytosis [32]. Optimal use of this pathway thus requires the presence of serum, or the addition of a lipopolysaccharide binding protein [32]. In agreement with these previous results, we found that uptake of BCG by hMDM in the absence of serum was poorly inhibited when the CR3 pathway was blocked. Increased uptake of r-BCG PGL-1 by hMDM was only observed in non-opsonic conditions. In addition, strong inhibition of r-BCG PGL-1 entry in hMDM was observed following CR3 blockade. These results are consistent with previous studies showing that *M. leprae* preferentially invades human monocytes through the CR3 receptor in non-opsonic conditions [29]. Our findings suggest that, in the absence of opsonins, PGL-1 interacts either with a co-receptor of the CR3 mediated phagocytosis pathway or more likely with CR3 itself. This interaction might occur through the lectin site of the CR3 alpha chain which was shown to bind various sugar moieties [33]. The terminal disaccharide of PGL-1, which is missing in PGL-bovis, may therefore be crucial for the interaction with CR3. Engagement of CR3 has been reported to be associated either with pro- or anti-inflammatory responses, depending on the ligand and costimuli [34]–[36]. For instance, the fungus pathogen, *Blastomyces dermatitidis* uses the CR3 phagocytosis pathway for TNF-α suppression and immune evasion [37]. Here we demonstrate that PGL-1 production confers similar properties to mycobacteria, as preferential phagocytosis of r-BCG PGL-1 *via* CR3 induced lower inflammatory responses than those observed with BCG. With regard to DC, *M. leprae* has been reported to inhibit their infection-induced cell maturation and subsequent release of proinflammatory cytokines by comparison to BCG, or *M. tuberculosis* [14]. *M. leprae*-infected DC showed defective expression of major histocompatibility complex II expression and CD83 costimulatory molecule, resulting in poor induction of CD4+ and CD8+ T cells responses [13]. Our observation that PGL-1, when expressed by BCG, suppresses the maturation of hDC strongly suggests that PGL-1 is responsible for the impaired maturation of *M. leprae*-infected hDC. On the basis of our results using human macrophages and dendritic cells, we propose that by promoting phagocytosis of *M. leprae* bacilli *via* CR3, PGL-1 expression may contribute to the defective cellular responses of multibacillary lepromatous leprosy patients.

In conclusion, we developed in this study an innovative approach to understand the role of PGL-1 in the leprosy pathogenesis. This approach might be extended to the study of PGL and lipids produced by other mycobacterial species. With regard to PGL, our knowledge of the biosynthesis pathway of PGL-1 and PGL-tb will largely facilitate the construction of BCG expressing PGL of other human pathogens. For instance, the saccharidic domain found in *M. marinum* and *M.ulcerans*, i.e. 3-*O*-Me-Rhap (α1- linked to phenol ring) [38], [39], corresponds to the first residue of the carbohydrate domain of PGL-1. Therefore, the microbial tools are now available to compare the biological properties of the various PGL in the context of comparable and relevant mycobacterial cell envelopes. This information is crucial to understand the specificities of the various mycobacterial diseases, such as the different organ tropism or subversion of host immunity.

## Materials and Methods

### Bacterial strains and growth conditions

*M. bovis* BCG Pasteur 1173P2 was cultured in Middlebrook 7H9 broth (Invitrogen, Cergy-Pontoise, France) containing ADC (0.2% dextrose, 0.5% bovine serum albumin fraction V, 0.0003% beef catalase) and 0.05% Tween 80 and on solid Middlebrook 7H11 broth containing ADC and 0.005% oleic acid (OADC) (Becton Dickinson, Sparks, USA). When required, kanamycin (Km) and hygromycin (Hyg) were added to the medium at the final concentration of 40µg/ml and 50µg/ml respectively.

### Construction of plasmids and mutant strains of *M. bovis* BCG

A 4.4kb DraI-NsiI fragment was recovered from cosmid B971 that contains a large portion of the *M. leprae* genome [40] and inserted within plasmid pMIP12H [24] to yield plasmid pBNF03. This plasmid carried open reading frames ML0126 to ML0128.

Plasmid pWM76 was generated by insertion of a 5.4 kb Bst1107-XbaI fragment from cosmid L518 (containing genes ML2346 to ML2348) between the AatII (previously blunt-ended)-NheI restriction sites of vector pMV361 [25].

Plasmid pWM122 was constructed by insertion of two PCR fragments between the NdeI and NheI sites of plasmid pMV361e, a derivative of pMV361 containing the mycobacterial promoter pBlaF* [25] [41]. The first PCR fragment, containing genes ML0126, ML0127 and ML0128, was obtained with primers 0126 (5′-ATACATATGAGAGCAGCCGAAGCTTC-3′) and 0128 (5′-ATAACTAGTGACACTCAATCCGGTCACC-3′), using plasmid pBNF03 as template DNA. The second PCR fragment, containing genes ML2346, ML2347 and ML2348, was amplified using primers 2346 (5′-TATAAGCTTCAATCCAGCCGGGCGTGT-3′) and 2348 (5′-ATATCTAGACGTGTAGTGTCCACCGTT-3′).

The mutant *M. bovis* BCG *ΔRv2959c* was constructed using the strategy described by Bardarov et al. [42]. Briefly, a PmeI fragment, containing the *Rv2959c* gene disrupted by a kanamycin cassette flanked by two *res* sites from transposon γδ, was obtained from plasmid pPET14 [16] and inserted between the XbaI-SpeI sites (made blunt) of cosmid pYUB854 [42]. The resulting cosmid was cut with PacI and ligated with the mycobacteriophage phAE87 to form the recombinant mycobacteriophage phWM06. Phage particules were then used to infect *M. bovis* BCG and allelic exchange mutants were selected on 7H11 agar plates supplemented with Km and OADC. Mutant clones were screened as previously described [16] and one clone was selected for further study. The unmarked mutant was generated following transient expression of transposon γδ resolvase from plasmid pWM19 [16]. One clone, PMM130, with an amplification pattern consistent with the excision of the kanamycin cassette was retained for further analysis (Figure S1). The various plasmids were transferred in *M. bovis* BCG or PMM130 by electrotransformation and transformants were selected on 7H11 agar plates supplemented with OADC and Hyg.

The various *M. bovis* BCG recombinant strains were rendered fluorescent by the transfer of plasmid pWM124, a derivative of the mycobacterial plasmid pMIP12H allowing expression of *gfp* gene from pblaF* promoter.

### Biochemical analysis of *M. bovis* BCG recombinant strains

PGL produced by the various *M. bovis* BCG recombinant strains were extracted and analyzed as previously described [24]. For quantification of the PGL production in WT BCG and r-BCG PGL-1, each strain was cultured in 7H9 supplemented with ADC and 0.05% Tween 80 to exponential growth phase and labeled with 0.625 µCi.ml^−1^ [1-^14^C] propionate (specific activity of 54 Ci.mol^−1^) for 24h. Lipids were extracted and analyzed as previously described [43]. To compare the amount of PGL-1 produced by r-BCG PGL-1 and *M.leprae*, 200 and 400 µg of total lipids extracted from r-BCG PGL-1 grown 20 days in 7H9 supplemented with ADC or *M. leprae* recovered from infected armadillos (kind gift from Dr P. J. Brennan and Dr J. S. Spencer from Colorado State University, Fort Collins, CO, USA) were spotted onto a silica gel 60 thin-layer chromatography (TLC) plate (20×20 cm, Merck). The TLC plate was run in CHCl_3_/CH_3_OH (95∶5, v/v) and PGL were visualized by spraying the plates with a 0.2% anthrone solution in concentrated H_2_SO_4_, followed by heating. Lipids were quantified with a CAMAG TLC scanner using the Win CATS v1.4.3 software.

### Sensitivity to oxygen or nitrogen radicals

Exponentially growing bacteria were diluted 1∶100 in fresh liquid medium containing various concentrations of H_2_O_2_ (0, 3, 6 or 12 mM) or NaNO_2_ at pH 5.5 (0, 2.5 or 5 mM for NaNO_2_) to generate NO and NO_2_^−^. After 24 h of incubation at 37°C with the chemicals, serial dilutions were plated on 7H11 solid medium. Cfus were counted after three weeks of incubation at 37°C. The experiments were performed three times independently.

### hMDM or hDC cultures

Human blood samples, purchased from the Etablissement Français du Sang of Toulouse (France), were collected from fully anonymous non-tuberculous control donors. Peripheral blood mononuclear leukocytes and hMDM were obtained as previously described [44]. Briefly, peripheral blood monocytes were cultured for 7 days on sterile glass coverslips in 24-well tissue culture plates (5×10^5^ cells/well) containing RPMI 1640 (Gibco, Cergy Pontoise, France) supplemented with 2 mM glutamine (Gibco) and 7% heat inactivated human AB serum. The culture medium was renewed on the third day. The hMDM were washed twice with fresh RPMI medium before use.

For hDC, peripheral blood mononuclear cells were isolated from whole blood by sedimentation over a Ficoll-Hypaque gradient (GE Healthcare) and monocytes purified by negative selection (Miltenyi Biotec). Immature DCs (iDCs) were prepared from this CD14^+^ fraction by culture in RPMI 1640 supplemented with 1% human serum (DC medium), in the presence of 1,000 U/ml GM-CSF (and 500–1,000 U/ml IL-4 (Peprotech) for 6 days. DC maturation was monitored by flow cytometry using APC-conjugated mouse anti-human CD83 (HB15e) or CD40 (5C3), PE-conjugated mouse anti-human CD80 (L307.4) or HLA-DR (G46-6), all from BD Biosciences. For infection studies, DCs were then plated in 96 well plates at a density of 100,000 cells per 200 µl in DC medium.

CR3-transfected CHO-Mac1 cells are CHO cells stably expressing human CR3. A subclone of CHO-Mac1 cells expressing high levels of CD11b/CD18 was used in our experiments. Cells were cultured in α-MEM supplemented with 10% heat-inactivated fetal bovine serum, L-glutamine and for CR3-transfected CHO cells 0.1 µM methotrexate (Sigma, St Louis). Prior to infection with mycobacteria, CR3 expression was verified with a PE conjugated anti-CR3 mouse monoclonal antibody (clone 2LPM) (Dako, Trappes, France) and analyzed by flow cytometry on a FACScalibur (Becton Dickinson) and cells were seeded (4×10^5^ per well) over 12 mm diameter glass coverslips in 24-wells plates and grown overnight at 37°C.

### Cell infections

Immediately before infection, mycobacteria grown to exponential phase were pelleted at 5000 g for 10min, washed twice in PBS, resuspended in serum free interaction medium (for experiments performed under non-opsonic conditions) or pre-incubated in fresh human AB serum for 30 minutes at 37°C (for experiments under opsonic conditions). Mycobacteria clumps were dispersed by drawing up and expelling the bacterial suspension 20 times through a 25G needle attached to a 1-ml syringe. After a clarification step, achieved by low speed centrifugation (200 g), efficiency and reproducibility of dispersal were checked for each strain by microscopic observation (magnification ×400). The resulting suspension was diluted 1∶10 in the interaction medium (supplemented with 2% of fresh human AB serum for experiments under opsonic conditions) and optical density was read at 600 nm versus relevant blanks. For all infection experiments with the WT and recombinant *M. bovis* BCG strains, we established that 0.1 OD unit corresponded to 1×10^7^ bacilli (this was checked by colony forming unit determination and confirmed by direct numeration of bacteria under microscopic observation in a Thoma chamber. Bacteria were further diluted in serum free interaction medium at the desired multiplicity of infection (MOI), as stated in figure legends. For each strain and each individual experiment, CFU numbers were determined by plating serial dilutions of bacterial suspension on 7H11 agar supplemented with 10% OADC.

When specified, bacilli were coated with purified PGL-1 or PGL-bovis by suspending 5×10^7^ bacteria in 100 µl of 0.05% PGL in petroleum ether as described previously [44]. Control was prepared by treating bacilli with solvent alone. The solvent was evaporated off and the bacteria were resuspended in PBS.

### Phagocytosis assays

Phagocytosis was assessed as previously described [44]. The role of MR and CR3 in phagocytosis by hMDM was evaluated by incubating cells with anti-CR3 (2LPM) or anti-MR monoclonal antibodies at 10 µg/mL before infection. An irrelevant isotype-matched antibody was used as control.

### Colocalization experiments with Lysotracker, v-ATPase or CD63

Maturation of phagosomes in hMDM was evaluated as previously described [44]. Recombinant WT BCG or r-BCG PGL-1 expressing the *gfp* were used for these experiments. Briefly, after infection, hMDM were washed and incubated with fresh medium. LysoTracker Red labelling was performed by washing hMDM at different time points after infection and incubating them with the acidotropic dye (1∶2000) in RPMI 1640 for 1 h. Rinsed cells were fixed with 3.7% paraformaldehyde for 1 h. For v-ATPase or CD63, mouse monoclonal Ab against human CD63 and rabbit polyclonal anti-serum against v-ATPase proton pump were obtained from Caltag Laboratories (Burlingame, USA) and from Synaptic Systems (Göttingen, Germany), respectively. Macrophages were fixed as described above, permeabilized by incubation with 0.3% Triton X-100 for 10 minutes at room temperature (RT), blocked by incubation with 0.3% BSA and incubated with antiserum against v-ATPase (1/100) or mouse anti-CD63 Ab (1∶100) for 1 h at RT, and revealed with Rhodamine-Red conjugated goat anti-rabbit or anti-mouse Ab. Colocalization of WT BCG or r-BCG PGL-1 with the various maturation markers was quantified with a Leica DM-RB fluorescence microscope. Colocalization was determined as the fraction of phagosomes with GFP fluorescence associated with LysoTracker, v-ATPase or CD63 markers. For each marker, 100 phagosomes from at least 10 different fields in duplicate in two independent experiments for each time points were counted.

### Quantification of NF-κB activity and TNF-α secretion in human phagocytes

The NF-κB activity resulting from cell stimulation with mycobacteria was studied using the THP-1 Blue-CD14 cell line (Invivogen, Toulouse, France). This cell line is a derivative of THP-1 (human monocyte/macrophage cell line) that over-expresses CD14 and is stably transfected with a reporter plasmid expressing a secreted embryonic alkaline phosphatase (SEAP) gene under the control of a promoter inducible by NF-κB and AP-1. Cells were cultured according to the manufacturer's instructions. Bacteria were deposited in 96-wells plates at the indicated concentrations in a volume of 20 µl and cells were added in 180 µl at 10^5^ cells per well in the HEK-blue detection medium (Invivogen) that contains a substrate for the SEAP and fetal calf serum. Alkaline phosphatase activity, corresponding to NF-κB activation, was measured after 16h by reading OD at 630 nm. Positive controls lipomanan and lipopolysaccharide induced consistent and relevant NF-κB activity. Unstimulated THP-1 cells had a marginal NF-κB activity representing 2% of the stimulation induced by WT-BCG. TNF-α secretion was assessed in supernatants with a quantitative ELISA test supplied by R&D (Abingdon, UK), according to the recommendations of the manufacturer.

### Statistics

Data are presented as the mean ± standard error of the mean (SEM) or standard deviation (SD) of the indicated number of independent experiments (n) performed in duplicates (phagocytosis assays) or triplicates (NF-κB activity detection or TNF-α secretion quantifications). The significance of differences was determined with the non-parametric test of Wilcoxon for paired samples to take into account the inter-donor variability in our analysis.

## Supporting Information

Figure S1 Construction of a recombinant *M. bovis* BCG strain producing PGL-1. A) Schematic representation of the genetic structure obtained during the construction of the *M. bovis* BCG *ΔRv2959c::km* and *M. bovis* BCG *ΔRv2959c::res* recombinant strains. The black box indicates the coding sequence. The light gray region represents the fragment deleted during the construction of the knock-out mutant. The dark grey box corresponds to the kanamycin (*km*) cassette flanked by the two *res* sites form transposon γδ. Binding sites of the primers used for PCR analysis are indicated. B) PCR analysis of the *M. bovis* BCG *ΔRv2959c::km* and *M. bovis* BCG *ΔRv2959c::res* recombinant strains. The recombinant and parental strains were characterized by PCR amplification using primers C (5′-ATGTGGAGAATGCTCTGCGCC-3′), D (5′-ACGTTCTTCAGGTGGTTCCGG-3′), E (5′-AACTCGCTCAGGATCTCCTGG-3′), res1 (5′-GCTCTAGAGCAACCGTCCGAAATATTATAAA-3′) and res2 (5′-GCTCTAGATCTCATAAAAATGTATCCTAAATCAAATATC-3′). PCR was carried out in a final volume of 50µl containing 1mM primers, 2.5 units of GoTaq DNA polymerase, 10% Me_2_SO according to the recommendation of the manufacturer (Promega, Charbonnieres, France). The amplification program consisted of 1 cycle of 2 min at 94°C followed by 30 cycles of 30s at 94°C, 30s at 60°C, 1min at 72°C and a final 10min at 72°C. kb, kilobases. C) Schematic representation of the genes carried by the various plasmids used for the construction of the various recombinant *M. bovis* BCG strains.
(1.11 MB EPS)

Figure S2 Effect of PGL-1 production on infection of hDC. Percentage of infected hDC after 2 h of contact with the mycobacterial strains at various MOI. The values are means ± SEM of 4 independent experiments. Black bars correspond to WT BCG and white bars to r-BCG PGL-1. For the four independent experiments, 100% corresponded to 56.3%, 41.6%, 20.2% and 43.9% of infected hDC. The significance of differences between BCG control and r-BCG PGL-1 was evaluated: *, p<0.05; n.s., not significant.
(0.47 MB EPS)

Figure S3 Impact of PGL-1 on IL-10 and IL-12 production by hMDM. hMDM were infected for 120 minutes with WT BCG (black bars) or r-BCG PGL-1 (white bars) at MOI 10, washed and further incubated in the presence of serum. At 2 h or 24 h post-infection, the culture supernatant was removed and IL-10 (A), IL-12p40 (B) or IL-12p70 (C) were assessed by ELISA. Values represented the mean + SEM of 2 independent experiments each performed in duplicate.
(0.68 MB EPS)
